# The effect of gestational age on mitochondrial properties of the mouse placenta

**DOI:** 10.1530/RAF-21-0064

**Published:** 2022-01-28

**Authors:** Lucy A Bartho, Joshua J Fisher, Sarah L Walton, Anthony V Perkins, James S M Cuffe

**Affiliations:** 1 School of Pharmacy and Medical Science, Griffith University, Gold Coast Campus, Southport, Queensland, Australia; 2 Hunter Medical Research Institute and School of Medicine and Public Health, University of Newcastle, Newcastle, New South Wales, Australia; 3 School of Biomedical Sciences, University of Queensland, St. Lucia, Queensland, Australia

**Keywords:** placenta, mitochondria, ageing, endoplasmic reticulum, senescence

## Abstract

Mitochondria are organelles within the cell that generate energy, which is essential to the developing placenta. As the placenta approaches term, organelles such as mitochondria and the endoplasmic reticulum adapt to cellular stressors (e.g. oxidative stress and fluctuations in oxygen concentration) which are likely to result in the progressive decline of tissue function, known as placental ageing. This ageing phenotype may induce cellular senescence, a process whereby the cell is no longer proliferating, yet remains metabolically active. Mitochondria, endoplasmic reticulum and senescent processes are still poorly understood in the developing placenta. Therefore, a rodent ontogeny model was used to measure genes and proteins involved in mitochondrial biogenesis, antioxidant function, electron transport chain, mitophagy, dynamics and unfolded protein response in the placenta. CD-1 mouse placental samples were collected at embryonic day (E)12.5, E14.5, E16.5 and E18.5 of pregnancy for gene and protein analysis via qPCR, protein assays and Western blotting. Mitochondrial content, SDHB (complex II) and MFN2 (mitochondrial fusion) proteins were all increased throughout pregnancy, while citrate synthase activity/mitochondrial content, *Tfam, Sirt3, Mfn1,* TOMM20 (mitochondrial biogenesis and dynamics);* Tp53*(senescence)*; Eif2ak3, Eif4g1*(endoplasmic reticulum stress)*;*NDUFB8, UQCRC2, ATP5A (electron transport chain sub-complexes) were decreased at E18.5, compared to E12.5. Overall, mitochondria undergo changes in response to gestational progression and pathways associated with cellular ageing to facilitate adaptions in a healthy pregnancy. This data holds great promise that mitochondrial markers across pregnancy may help to establish when a placenta is ageing inappropriately.

**Lay summary:**

Human pregnancy lasts approximately 266 days. If a baby is born early, organs may be poorly formed but if pregnancy continues past this time, stillbirth risk is increased. Gestational duration is regulated by the placenta. As the placenta approaches the end of pregnancy, it displays properties similar to tissues from aged individuals. However, it is unknown how this placental ageing contributes to pregnancy duration. This study characterised normal placental ageing by measuring properties of mitochondria in healthy placentas collected at four different gestational ages ranging from 7 days before birth to 1 day before birth of the 19-day mouse pregnancy. We found that mitochondrial number increased per cell but that a marker of mitochondrial function was reduced. Proteins that control mitochondrial number, morphology and function also changed over time. This work lays the platform to understand how placental ageing contributes to adverse pregnancy outcomes related to altered pregnancy duration.

## Introduction

Mitochondria are essential organelles that play a vital role in the generation of energy in all tissues, including the placenta (Bustamante *et al.* 2014). As the placenta develops, mitochondrial populations must adjust to fluctuations in oxygen concentration and apoptotic signals (Lu & Sferruzzi-Perri 2021). Mitochondrial adaptation involves processes such as fusion and fission also known as mitochondrial dynamics, as well as formation of new mitochondria (biogenesis) and mitophagy, the mitochondrial equivalent of autophagy (Bartho *et al.* 2020). Mitochondrial fusion results in interconnected mitochondria that have increased metabolic activity, while fission results in smaller, fragmented mitochondria which are less metabolically active (Westermann 2010). Dynamic mitochondrial adaptions likely influence, and are influenced by, the chronological ageing of the human placenta and if any of these mechanisms do not occur, or are dysfunctional across gestation, it is likely to result in placental insufficiencies and poor fetal development.

Mitochondrial dysfunction is linked to various aspects of biological ageing, such as impaired oxidative phosphorylation (OXPHOS) (Muralimanoharan *et al.* 2012), reduced antioxidant capacity, changes in mitochondrial dynamics (Mishra & Chan 2014) and mitochondrial quality control mechanisms. Therefore, an intricate balance between mitochondrial dynamics, biogenesis and mitophagy is critical in maintaining cellular homeostasis during tissue ageing (Gureev *et al.* 2019). Mitophagy prevents mitochondrial fusion and acts by removing dysfunctional mitochondria from the cell. Defective mitophagy have been linked with age-related disorders, such as Parkinson’s and Alzheimer’s disease (Ferree & Shirihai 2012). However, mitophagy has not yet been measured in the context of placental ageing.

Specific regulation of ageing influenced by mitochondria can be observed through damage to the electron transport chain complexes (ETC). Dysfunction in the ETC has been shown to initiate cellular senescence, a process identified in ageing tissue where the cell no longer proliferates but remains metabolically active (Zorova *et al.* 2018). Cells that are in a senescent state often display numerous smaller mitochondria that have likely undergone mitochondrial fission (Westermann 2010). Evidence of trophoblasts becoming senescent towards the end of pregnancy has been demonstrated with increased p53 signalling, endoplasmic reticulum (ER) stress and reactive oxygen species (ROS) signalling when labour is approaching, although the relationship between senescence and mitochondria in the placenta is still largely unknown (Cox & Redman 2017).

Excessive ROS production is associated with mitochondrial dysfunction, but given that ROS production is also increased when cells become senescent, it is likely that a link between these two factors exists. ROS are intrinsically linked to increased calcium efflux and the activation of proapoptotic proteins, prompting cell death through the mitochondrial permeability transition pore (Ott *et al.* 2007). The primary storage site for calcium in the cell is the ER; however, mitochondria and the ER work in a bidirectional manner to regulate calcium flux, mitochondrial dynamics and bioenergetics through mitochondrial–ER-associated membranes. The mechanisms behind increases in cellular stress and the downstream effects on the mitochondrial/ER unit throughout biological changes in pregnancy are unknown.

Factors that influence mitochondrial function in ageing tissue include proteins that regulate fusion and fission including mitofusins 1 and 2 (MFN1 and MFN2) (Chen *et al.* 2003). Other proteins involved in the regulation of mitochondrial dynamics include the family of proteins known as sirtuins. Sirtuins are NAD-dependent deacetylases that are essential for metabolic homeostasis and play a role in longevity through acting as molecular sensors of cellular energy balance and responding to metabolic changes in nutrient availability (He *et al.* 2012).

The placenta is the primary transient organ that facilitates the development of the fetus. The placenta only exists for a finite time of approximately 40 weeks and must undergo constant adaptation to adjust to the increasing demands of the developing fetus. Placental mitochondria play a major role in mediating these changes in placental function, although how factors involved in mitochondrial dynamics, biogenesis, mitophagy and the mitochondrial–ER relationships change across gestation remain unknown. This is in part due to the difficulty of safely collecting tissue throughout pregnancy and the ethical regulations associated with such a study in humans. As such, a mouse ontogeny model of pregnancy was designed to assess normal physiological aging processes within the placenta across the second half of the 19-day gestational period of the mouse. Given that the mature mouse placenta is established at E10.5, the current study aimed to investigate how mitochondrial processes associated with ageing change from this stage of gestation until close to term (Woods *et al.* 2018).

## Methods and materials

### Ethical approvals

All animal experiments were approved by the University of Queensland Animal Ethics Committee (AEC 484/09 and 496/12) and were conducted in accordance with the Australian Code of Practice for the Care and Use of Animals for Scientific Purposes. Animal procedures, including mating, feeding and tissue collection have been previously described (Cuffe *et al.* 2014). Pregnant, untreated CD-1 mice were sacrificed at embryonic day (E) 12.5, E14.5, E16.5, E18.5 by cervical dislocation for the collection of whole placentas. Placentas were randomly selected from three litters, per age (E12.5, E14.5 and E16.5) and six litters (E18.5). Additionally, fetal tails were collected for DNA extraction and fetal sex was determined using PCR amplification of SRY for all placentas from the litters used in this study. Following sex determination, an equal number of males and females were selected to eliminate any bias in outcomes due to one sex over the other. From these samples, two males and two females per litter were selected for analysis. These samples were randomly selected from throughout the litter to ensure that the position within the uterine horn did not impact results. By including multiple animals per litter, the impact of horn position (and therefore blood flow/oxidative stress patterns) on placental outcomes was minimised. The tissue was collected and frozen for qPCR (*n* = 10, five per sex, from three to six litters) and Western blot analysis (*n*  = 6, three per sex, at E12.5, E14.5, E16.5 and E18.5, from three to six litters).

### mRNA gene expression studies

Expression of genes involved in mitochondrial processes was measured by qPCR. RNA was extracted from placental tissue using the RNeasy minikit (Qiagen). RNA concentration was measured via absorbance spectrometry using the Nanodrop 2000/2000c. RNA was converted to cDNA using the iScript gDNA Clear Synthesis Kit (BioRad) according to the manufacturer’s protocol. RNA was normalised to 1000 ng/µL and reverse transcribed in a 10 µL reaction containing 2 µL of iScript RT Supermix. The RT reaction was performed as follows: priming at 25°C for 5 min, RT at 46°C for 20 min, deactivation of reaction mix at 95°C for 1 min. All PCR reactions were performed as per the MIQE guidelines (Bustin *et al.* 2009). Quantitative PCR was performed on the StepOne real-time PCR system (Applied Biosystems) with thermocycling parameters: initial activation step 95°C for 2 min, followed by 40 cycles of denaturation at 95°C for 5 s and combined annealing/extension at 60°C for 10 s. KiCqStart SYBR green PCR primers (Sigma–Aldrich) indicated in Table 1 were used to measure mRNA expression of genes. There was no product detected in the non-template control, and a single spike was indicated in the melt curve analysis. All gene expression was normalised to the mean of 18S which was stably expressed between timepoints. Additional housekeeper genes were assessed for suitability but were not selected due to differences in expression between timepoints. The final expression was calculated using the 2^−ΔΔCt^ method, and eight samples per group were used and run in duplicate.

**Table 1:** Quantitative mouse PCR primer list.

Gene name	Gene acronym	Accession number	Primer sequences	
			Forward	Reverse
18S ribosomal RNA	*18S*	NR_003278	CAGTTATGGTTCCTTTGGTC	TTATCTAGAGTCACCAAGCC
Transcription factor A, Mitochondrial	*Tfam*	NM_009360.4	GACCTCGTTCAGCATATAAC	ACAAGCTTCAATTTTCCCTG
Mitochondrial D-loop	*Dloop*	NC_005089	AATCTACCATCCTCCGTGAAA	GCCCGGAGCGAGAAGAG
NADH dehydrogenase subunit 2	*Nd2*	NP_904329.1	CACGATCAACTGAAGCAGCAA	ACGATGGCCAGGAGGATAATT
Beta actin	*ActB*	NM_007393.5	AGCCATGTACGTAGCCATCCA	TCTCCGGAGTCCATCACAATG
Glyceraldehyde-3-phosphate dehydrogenase	*Gapdh*	NM_001289726.1	AAGGTCATCCCAGAGCTGAA	CTGCTTCACCTTCTTGA
PTEN-induced kinase 1	*Pink1*	NM_026880.2	ACTTACAGAAGATCCAGAGATG	CTTCATAACGAGGAACAGTG
E3 ubiquitin-protein ligase parkin	*Park2*	NM_001317726.1	GAGAAGAGCAGTACACTAGG	CATGGTATGCTTCCTTACAG
Cellular tumour antigen p53	*Trp53*	Q549C9	TAGGTAGCGACTACAGTTAG	GGATATCTTCTGGAGGAAGTAG
Eukaryotic translation initiation factor 2-alpha kinase 3	*Eif2ak3*	Q9Z2B5	GAAGCCCTTAATCCATTCTC	GCACCATTACTGTATTCTTCG
Eukaryotic translation initiation factor 4 gamma 1	*Eif4g1*	Q6NZJ6	GCAATTATCTTTGAGACTCCTC	ACCACGTCCTCATCATATAG
Inositol 1,4,5-triphosphate receptor, type 3	*Itpr3*	P70227	TACTCTTCTGGATCCTCATC	CTTGTTGGTCAGATTGAGAG

### Mitochondrial content

Mitochondrial content was assessed to indicate how mitochondrial number changes across gestation. Relative mitochondrial content was assessed by dividing the average concentration of mitochondrial DNA (mtDNA) in the sample by the nuclear DNA (nDNA) in the sample. QPCR was utilised to amplify total extracted DNA (*n* = 10, five per sex, three to six litters per group) using primer sets for two mitochondrial-encoded DNA markers (*Dloop* and *Nd2*) and two nuclear-encoded DNA markers (*ActB*and *Gapdh*) (Refer to Table 1). The 2^−ΔΔCt^ method was used to normalise relative levels of the geometric mean of the two mitochondrial genes to the geometric mean of the two nuclear genes.

### Citrate synthase activity

Citrate synthase (CS) activity was measured as per a published protocol (Eigentler *et al.* 2015) as an indicator of mitochondrial activity. Whole tissue placental protein lysates were assayed (*n* = 6, three per sex, three to six litters per group) in duplicate across a single plate. Samples and standards were loaded into a 96-well plate in triplicate, and a reaction mix containing Triton X-100, acetyl coenzyme A and 5’5’-dithiobis (2-nitrobenzoic acid) was added. Oxaloacetate was added to initiate the reaction, and immediately after, absorbance was measured at 450 nm. Samples were normalised to protein concentration of 1 µg/µL, and CS activity was calculated as previously described (Eigentler *et al.* 2015).

### SOD activity

Superoxide dismutase (SOD) capacity was measured using a commercially available kit to quantify enzyme activity (Cayman Chemicals) as per the manufacturer’s instructions. Protein lysates were assayed in duplicate (*n* = 8, three to six litters per group) in a 96-well clear plate, and enzyme activity was normalised to protein concentration per sample. Absorbance changes were measured on a SpectraMax M4 Multi-Mode Microplate Reader (Molecular Devices, LLC). Enzyme activity was measured at an absorbance of 440–460 nm, and the final value was expressed as U/mg of protein.

### Western blotting

Mitochondrial dynamics allow for complex mitochondrial networks which may be independent of mitochondrial DNA content. As such, the expression of different components of mitochondrial complexes was assessed. Furthermore, the expression of proteins involved in the regulation of mitochondrial dynamics was assessed. Protein lysates were extracted from mouse placental tissue collected from E12.5, E14.5, E16.5 and E18.5. Protein concentrations were measured using the Pierce™ BCA Protein Assay Kit (ThermoFisher Scientific). 10 µg of protein was loaded per well into 12% polyacrylamide gels for separation. Proteins were then transferred onto low fluorescence polyvinylidene fluoride membranes. Membranes were blocked with Odyssey blocking buffer (Millipore) and incubated overnight at 4°C with primary antibodies raised in rabbit: tumour protein 53 (P53-1:1000, Abcam, ab131442), transcription factor A mitochondrial (TFAM – 1:1000, ab131607), translocase of outer mitochondrial membrane 20 (TOMM20-1:1000, ab186735). Following incubation, membranes were incubated with LICOR secondary antibodies anti-rabbit (IRDye 680 goat, Licor, Lincoln, NE, USA) 1:20,000 dilution and anti-mouse (IRDye 800 donkey, Licor, Lincoln, NE, USA) 1:20,000 dilution. The internal housekeeper used throughout the Western blotting was β-actin (1:1000, ab8227). The blots were imaged by Licor Odyssey and quantified by Image studio v 5.2.

### Statistical analysis

All data were analysed using GraphPad Prism v.9.0 and are presented as mean ±s.e.m. with the Grubbs test for outliers performed. Normality and lognormality of the data were assessed using the Shapiro–Wilk test. While data were initially analysed separately for males and females, no sex differences were detected and so both sexes were pooled and reanalysed with males and females combined. A one-way ANOVA with Tukey’s pairwise comparison was used when the data were normally distributed, and a Kruskal–Wallis test with Dunn’s pairwise comparison was selected when the data were not normally distributed to assess the differences between placentas collected at embryonic (E) day, E12.5, E14.5, E16.5 and E18.5 of pregnancy (*n*  = 10 per group, five per sex, from three to six litters). If a statistical difference was detected by ANOVA, then a suitable multiple comparison test was performed. *P*< 0.05 was considered significant in all assays, qPCR and Western blotting data.

## Results

Fetal body weights increased with advancing gestational age between E12 and E18.5 (*P*< 0.0001, Table 2). Placental weight also increased with advancing gestational age from E12.5 to E18.5 (*P*< 0.0001, Table 2).

**Table 2:** Comparison of fetal body weights and placental weights from mouse samples (*n* = 10) collected at embryonic (E) day E12.5, E14.5, E16.5, E18.5. Analysis by one-way ANOVA with Dunnett pairwise comparison. Data are presented as mean ± s.e.m.

	E12.5	E14.5	E16.5	E18.5	*P*
Fetal body weight (mg)	101.3 ± 2.84	308.8 ± 10.56	737.9 ± 15.22	1335 ± 31.51	<0.0001
Placental weight (mg)	59.74 ± 2.972	98.24 ± 3.53	114.5 ± 4.299	100.2 ± 3.99	<0.0001

### Ontogeny of mitochondrial function, content and antioxidant capacity

**Figure 1:**
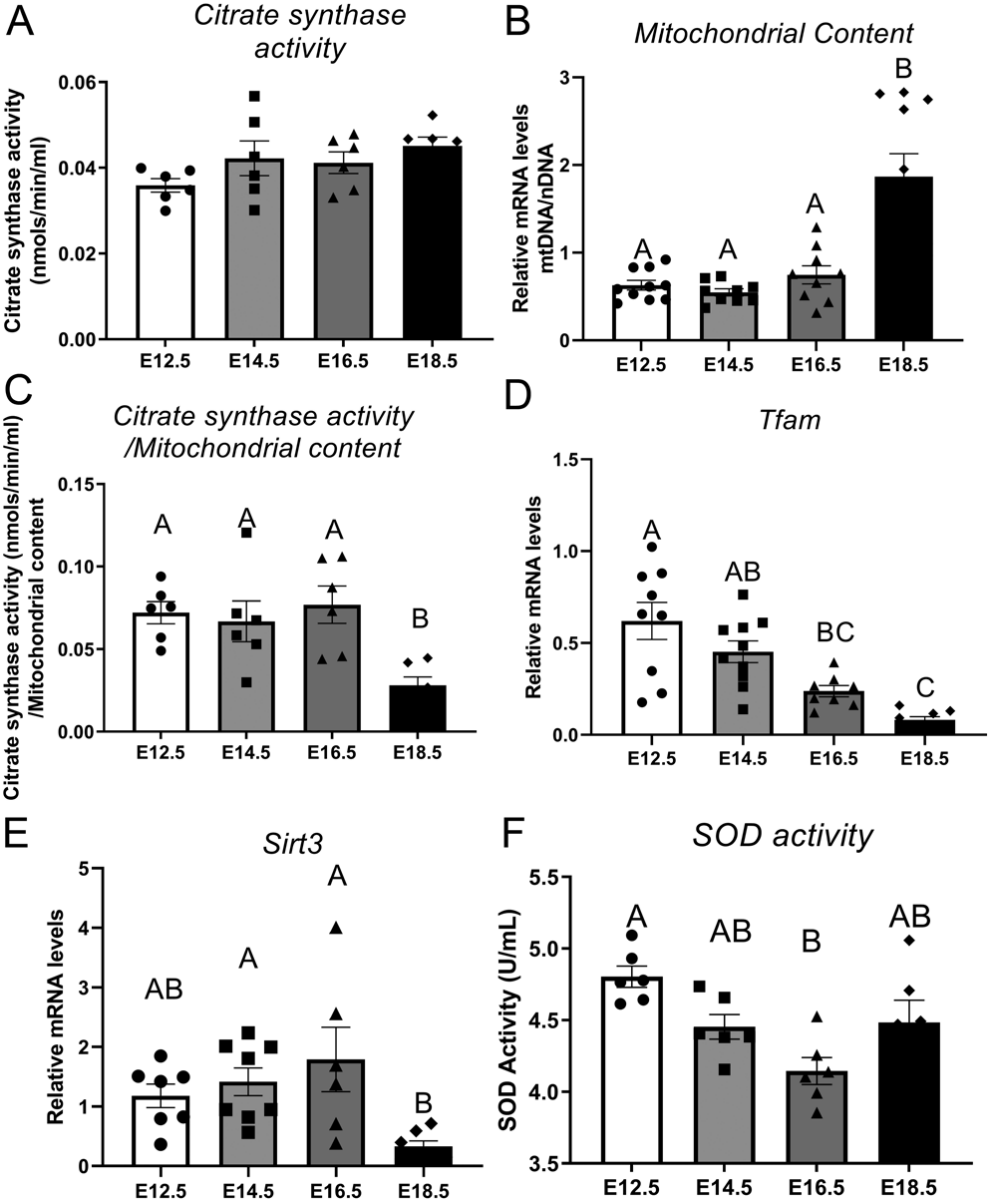
Citrate synthase activity (A), mitochondrial content (B), citrate synthase activity/mitochondrial content (C), mRNA expression of TFAM (D), SIRT3 (E) and SOD (F) activity of mouse placental samples collected at E12.5, E14.5, E16.5 and E18.5 (from three to six litters per gestational age). Significance was indicated when *P*< 0.05. Significant differences between groups as detected by *post hoc* analysis are denoted by different letters where A is different from B which is different from C. A is not different from A or AB, while B is not different from B, AB or BC. Data are presented as mean ± s.e.m. (*n* = 10, five per sex).

To understand how mitochondrial processes change within placental tissue across the second half of pregnancy, mitochondrial CS activity, mitochondrial content and SOD activity were measured within mouse placentas collected from E12.5, E14.5, E16.5 and E18.5. Similarly, markers of mitochondrial biogenesis were assessed. CS activity was unaffected by gestational age (Fig. 1A). Mitochondrial content, as determined by mtDNA/nDNA ratio, was affected by gestational age (*P* < 0.0001) with stable expression between E12.5 and E16.5 (Fig. 1B) but an almost three-fold increase between E16.5 and E18.5 (*P*< 0.0001). CS activity/mitochondrial content ratio was also impacted by gestational age with stable levels between E12.5 and E16.5 (Fig. 1C) but a significant decrease in placentas collected at E18.5 (*P* = 0.0055) compared to other timepoints. *Tfam* expression was consistent between E12.5 and E14.5 (Fig. 1D) and decreased with age thereafter (*P*< 0.0001). Placental *Sirt3*expression did not change from E12.5 to E16.5 (Fig. 1E) but was decreased at E18.5, when compared to E14.5 (*P* = 0.01) and E16.5 (*P* = 0.029). SOD activity was significantly decreased between E12.5 and E16.5 (*P*= 0.0016) (Fig. 1F) however remained unchanged between other groups.

### Adaptions of mitochondrial ETC complexes across gestation

**Figure 2:**
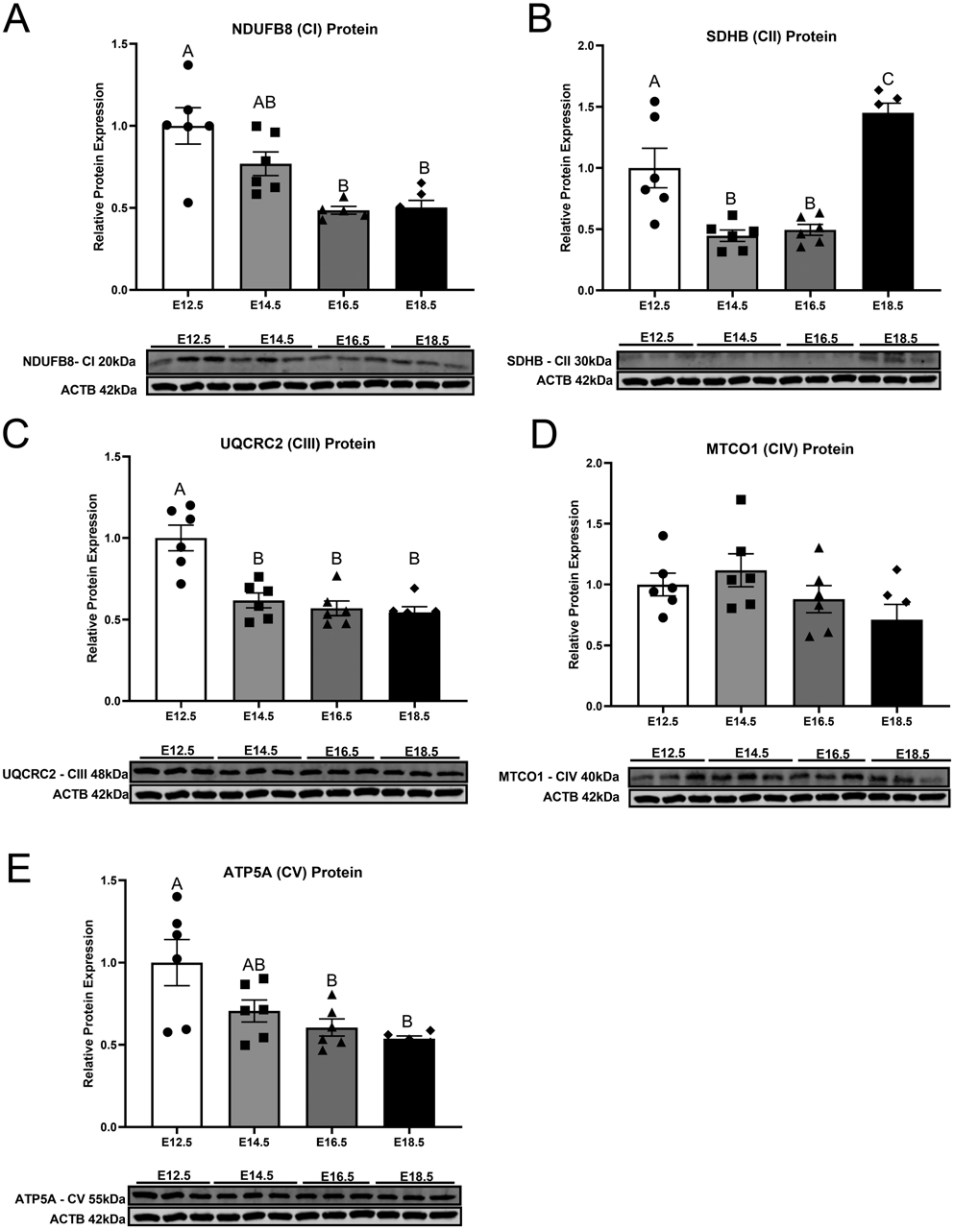
Protein expression of NDUFB8 (complex I) (A), SDHB (complex II) (B), UQCRC2 (complex III) (C), MTCO1 (complex IV) (D), ATP5A (complex V) (E) of mouse placental samples collected at E12.5, E14.5, E16.5 and E18.5 (from three to six litters per gestational age). Significance was indicated when *P*< 0.05. Significant differences between groups as detected by *post hoc* analysis are denoted by different letters where A is different from B which is different from C. A is not different from A or AB, while B is not different from B, AB or BC. Data are presented as mean ± s.e.m. (*n* = 6, three per sex).

To investigate how placental mitochondrial proteins change in abundance over the second half of mouse pregnancy, key proteins indicative of individual complexes from the ETC were measured. Subunit NDUFB8 of complex I was decreased between E12.5 and E16.5 *(P*= 0.0006) (Fig. 2A) and remained consistent thereafter. Subunit SDHB of complex II was reduced after E12.5 (*P*= 0.003) (Fig. 2B) and remained unchanged until a significant increase at E18.5 (*P*< 0.0001). Subunit UQCRC2, a marker of complex III in the ETC, was found to be highly expressed early in pregnancy (Fig. 2C) and decreased at E14.5–E18.5 *(P*= 0.0003*)*. Subunit MTCO1 of complex IV was not affected by gestational age (Fig. 2D). Expression of subunit ATP5A, a marker of complex V in the ETC, was lower at E16.5 *(P*= 0.017*)* and 18.5 *(P*= 0.007*)* (Fig. 2E), compared to E12.5.

### Ontogeny of mitophagy and senescence markers

**Figure 3:**
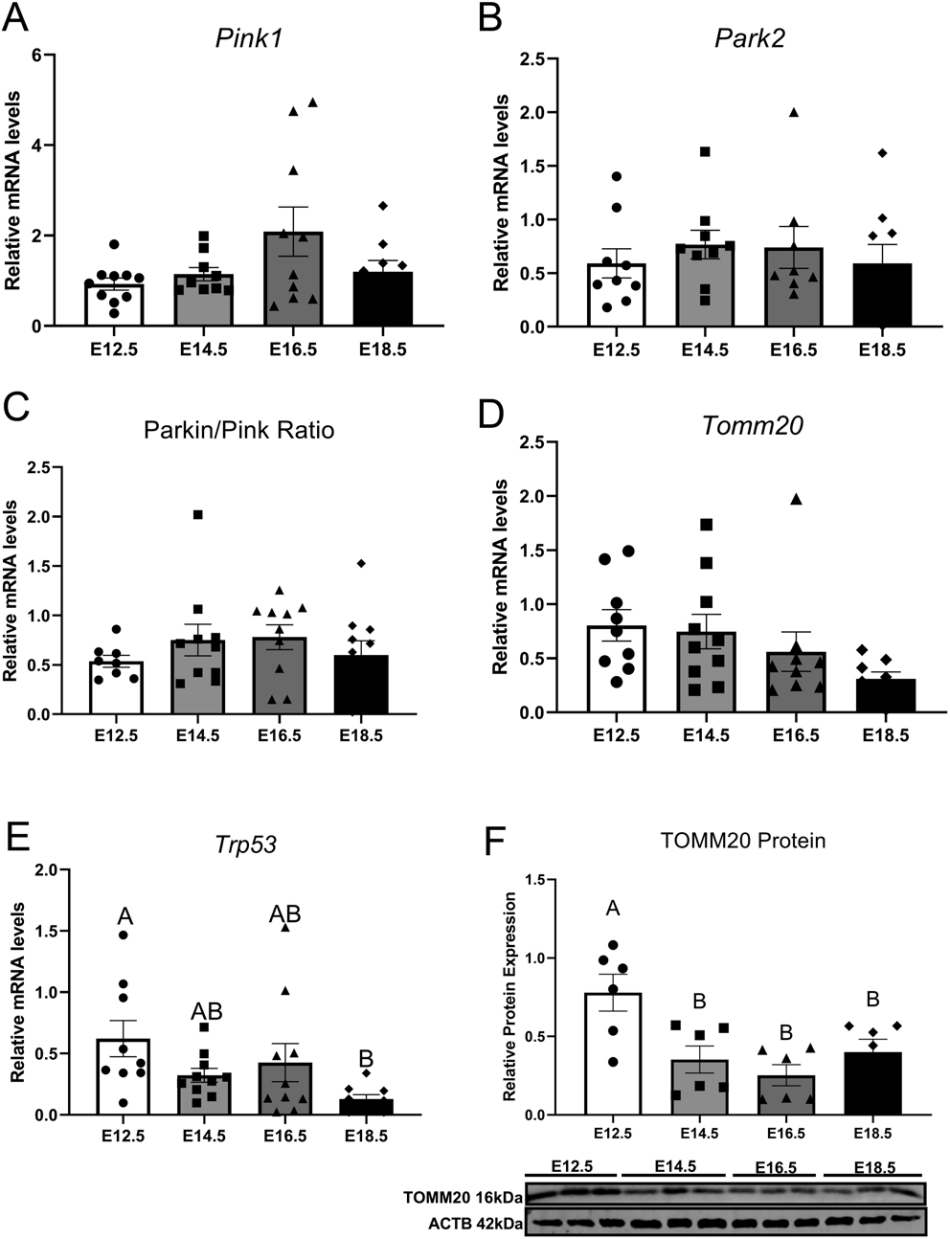
mRNA expression of Pink1 (A), Park2 (B), Parkin/Pink ratio (C), Tomm20 (D), Trp53 (E) and protein expression or TOMM20 (F) of mouse placental samples collected at E12.5, E14.5, E16.5 and E18.5 (from three to six litters per gestational age). Significance was indicated when *P*< 0.05. Significant differences between groups as detected by *post hoc* analysis are denoted by different letters where A is different from B which is different from C. A is not different from A or AB, while B is not different from B, AB or BC. Data are presented as mean ± s.e.m. (*n* = 10, five per sex for gene analysis and *n*  = 6, three per sex for protein analysis).

Placental mRNA transcripts for *Pink1, Park2, Tomm20*and *Trp53,*as well as TOMM20 protein expression, were detected at all timepoints examined throughout this study. Gestational age did not affect *Pink1*(Fig. 3A), *Park2*(Fig. 3B), *Parkin/Pink r*atio (Fig. 3C) and *Tomm20*(Fig. 3D) gene expression. Gene expression of *Trp53* was unchanged between E12.5 and E16.5 (Fig. 3E), although decreased at E18.5 compared to E12.5 (*P*= 0.024). Placental TOMM20 (Fig. 3F) protein expression was significantly lower throughout E14.5–E16.5 when compared to E12.5 gestational age (*P*< 0.0001).

### Ontogeny of mitochondrial fusion gene and protein expression

**Figure 4:**
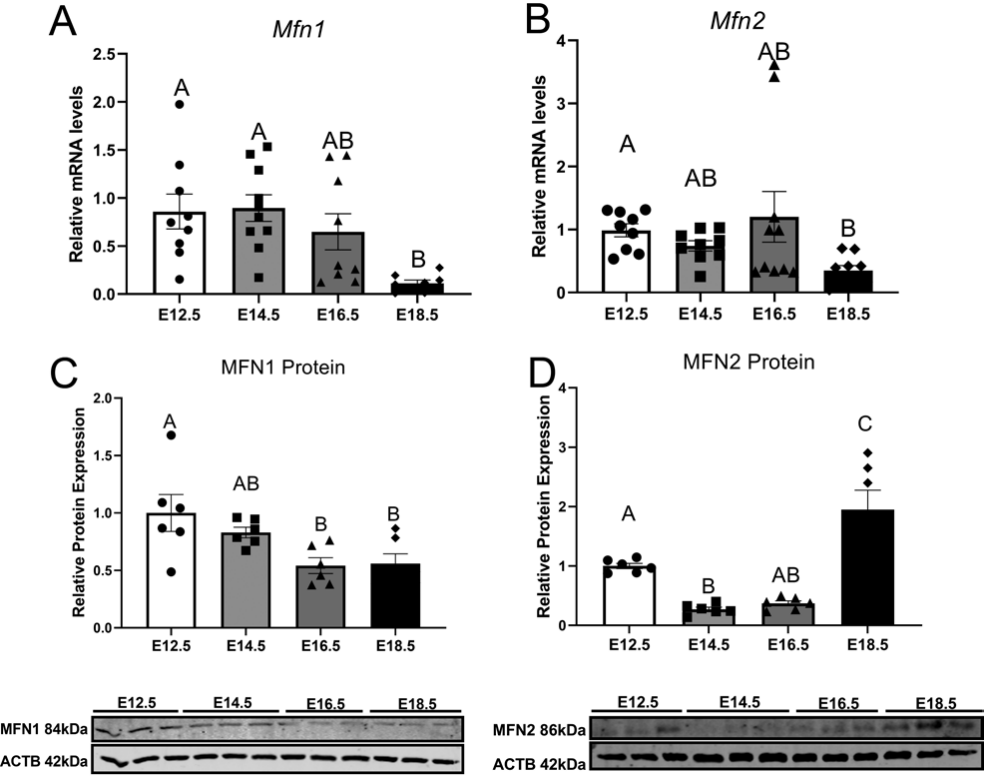
mRNA expression of Mfn1 (A), Mfn2 (B) and protein expression of MFN1 (C), MFN2 (D), of mouse placental samples collected at E12.5, E14.5, E16.5 and E18.5 (from three to six litters per gestational age). Significance was indicated when *P*< 0.05. Significant differences between groups as detected by *post hoc* analysis are denoted by different letters where A is different from B which is different from C. A is not different from A or AB, while B is not different from B, AB or BC. Data are presented as mean ± s.e.m. (*n* = 10, five per sex for gene analysis and *n*  = 6, three per sex for protein expression).

Mitochondrial fusion markers MFN1 and MFN2 were measured at a gene and protein level at all timepoints assessed within this study. mRNA expression of *Mfn1* did not change between E12.5 and E16.5 (Fig. 4A) but decreased at gestational age E18.5 (*P*= 0.0105). *Mfn2* gene expression is unaffected between E12.5 and E16.5 (Fig. 4B) before decreasing at E18.5 (*P*= 0.0052*)*compared to E12.5. Protein expression of MFN1 protein did not change between E12.5 and E14.5 (Fig. 4C) but was lower at E16.5 (*P*= 0.019) and E18.5 (*P*= 0.025*)* compared to E12.5. Gestational age affected MFN2 protein expression (Fig. 4D), which was decreased at E14.5 compared to E12.5 (*P*= 0.027) and remained stable until E18.5 where there was a significant increase of protein expression (*P*= 0.003).

### Ontogeny of ER unfolded protein response receptors

**Figure 5:**
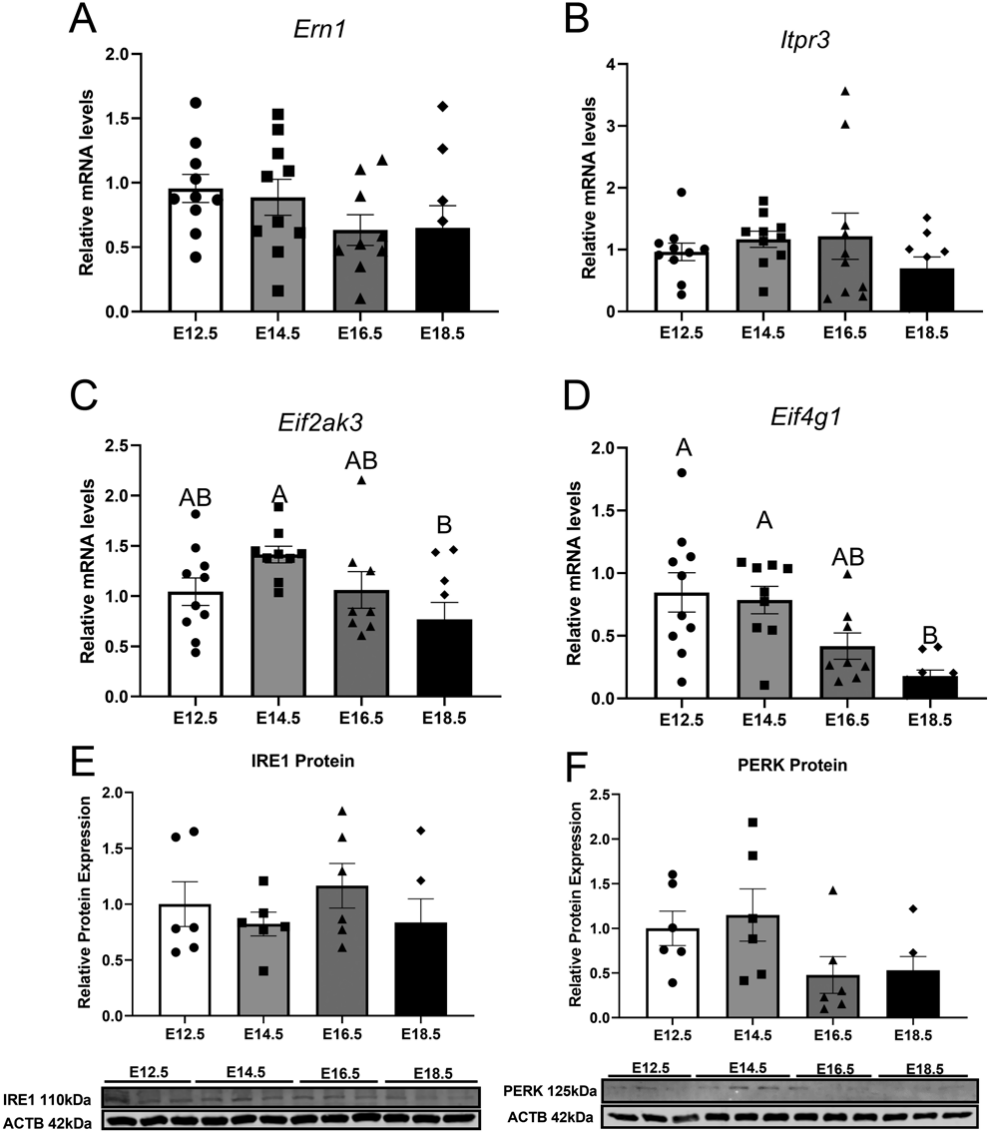
mRNA expression of Ern1 (A), Itpr3 (B), Eif2ak3 (C), Eif4g1 (D) and protein expression of IRE1 (E) and PERK (F) of mouse placental samples collected at E12.5, E14.5, E16.5 and E18.5 (from three to six litters per gestational age). Significance was indicated when *P*< 0.05. Significant differences between groups as detected by *post hoc* analysis are denoted by different letters where A is different from B. A is not different from A or AB, while B is not different from B or AB. Data are presented as mean ± s.e.m. (*n* = 10, five per sex for gene analysis and *n*  = 6, three per sex for protein analysis).

Expression of ER unfolded protein response receptors was measured in placentas collected between E12.5 and E18.5. Placental mRNA transcripts for *Ern1* (Fig. 5A) and *Itpr3* (Fig. 5B) were not affected by increased gestational age. *Eif2ak3* mRNA expression did not change between E12.5 and E14.5 (Fig. 5C); however, there was a significant decrease between E14.5 and E18.5 (*P*= 0.021). Placental gene expression of *Eif4g1*was not altered until E18.5 (Fig. 5D) when expression was significantly decreased when compared to E12.5 and E14.5 (*P*= 0.0012). Protein expression of IRE1 (Fig. 5E) and PERK (Fig. 5F) was not affected by gestational age.

## Discussion

While it is well-known that mitochondria play an important role in fetal development and are a contributing factor to pregnancy disorders (Bartho *et al.* 2020, Fisher *et al*. 2020), changes to mitochondria and ER across gestation in a normal, healthy pregnancy remain largely uncharacterised. This study is the first of its kind to investigate how mitochondrial properties change within the placenta across the second half of mouse gestation. Although mitochondrial function has been assessed in early human second trimester of pregnancy by Holland *et al.*, these samples are typically difficult to obtain across multiple timepoints of pregnancy, which would reduce the likelihood of identifying any subtle changes in mitochondrial biogenesis and energetics (Holland *et al.* 2017). In this current study, specific markers central to mitochondrial biogenesis, antioxidant function, ETC, mitophagy, dynamics and UPR were examined at gestational timepoints E12.5, E14.5, E16.5 and E18.5, equivalent to mid to late gestation in human pregnancy. Our results demonstrate that many of these indicators of mitochondrial function change as gestation advanced towards term. This further supports the dynamic nature of placental mitochondria and lays a foundation for future work interrogating how ageing processes may impact on placental function and hence fetal development.

A major finding of the current study was that although mitochondrial DNA content increased towards term, the expression of proteins located within different mitochondrial complexes decreased towards term. Furthermore, while total CS activity was stable across these four gestational timepoints, the CS activity to mitochondrial content ratio was significantly decreased at E18. This would support previous studies which have suggested that as cells become increasingly senescent, individual mitochondria become smaller and less metabolically active. It is likely that the increasing mitochondrial content is an important adaptation to advancing gestation to maintain mitochondrial function as the placenta is preparing for the end of pregnancy. Interestingly, expression of *Tfam*, a key regulator of mitochondrial DNA replication, decreased towards term. A finding that would suggest that as pregnancy neared full term, regulators of new mitochondrial formation decline to prevent excess mitochondria from forming. A mouse ontogeny model comparing mitochondrial respiration, substrate use, biogenesis and efficiency also found reduced CS as pregnancy progressed (Sferruzzi-Perri *et al.* 2019). In addition, mitochondrial biogenesis regulator peroxisome proliferator-activator receptor gamma coactivator-1-alpha showed a decreasing trend in expression with gestational age, which is concurrent with this current study’s findings (Sferruzzi-Perri *et al.* 2019). However, unlike this current study, these changes were localised to the junctional zone of the placenta, therefore suggesting that mitochondrial efficiency increases as the pregnancy approaches term (Sferruzzi-Perri *et al.* 2019). This is in contrast to other findings in aged rat brain which found decreases in *Tfam* expression are associated with decreases in mtDNA (Picca *et al.* 2013). Our data may be indicative of oversaturation of mitochondria per cell; therefore, there is no demand for increases in biogenesis and new mitochondrial DNA formation. Importantly, the current study also found that *Sirt3*, another indicator of mitochondrial biogenesis, decreased towards term. Multiple studies have indicated that CS, *Tfam* and *Sirt3* all decrease as tissues become aged which supports the idea that placental tissue close to term has mitochondria similar to those in aged adult tissue (Kwon *et al.* 2015, Woods *et al.* 2018).

To understand the normal progression of oxidative stress in ageing tissue, we assessed antioxidant responses during pregnancy via SOD activity, the enzyme responsible for converting the highly damaging superoxide to hydrogen peroxide (Wang *et al.* 2018). We identified a small but significant decrease in *SOD* activity at E16.5 compared to other timepoints which may be indicative of an increase in placental ROS (Rani *et al.* 2010). Previously, increased superoxide generation has been associated with decreases in SOD activity in placental trophoblast cells complicated by preeclampsia (Wang & Walsh 2001). Although this study measures SOD in a healthy pregnancy, decreases in SOD activity at E16.5 may also indicate ROS-based signalling in preparation for birth. Although this is yet to be demonstrated, future studies will focus on assessing the role of different SOD isoenzymes in the developing placenta.

OXPHOS is the final process by which REDOX reactions result in the generation of ATP. This study measured the protein expression of each complex in the ETC across pregnancy. Complex subunits NDUFB8 (complex I), UQCRC2 (complex III) and ATP5A (complex V) decreased from the start until the end of pregnancy, most likely due to the higher number of mitochondria functioning at a lower capacity, suggesting progression to a senescent phenotype. However, this study found that SDHB (complex II) is increased at E18.5 when compared to the other timepoints measured. Unlike the other complexes, complex II is a component of the citric acid cycle (TCA) where succinate is oxidised to fumarate. Throughout this process, complex II directly receives FADH_2_ therefore bypassing complex I and as a result, is less efficient at producing ATP (Cecchini 2003). However, in a study by Tatarkova *et al.*, complex II activity was also less impacted by aging in cardiomyocytes in comparison to complex I (Tatarkova *et al.* 2018). Additionally, research by Salazar-Petres *et al*. suggests that changes to the mitochondrial phenotype, including alterations in mitochondrial complex oxygen consumption rates, may be varied based on fetal sex and weight within each litter (Salazar-Petres *et al.* 2021). Although, this current study did not separate the data based on sex, this increase in complex specific proteins may be reflective of the metabolic shift nearing the end of pregnancy, although more research is required to elucidate this mechanism.

Given our results of an abundance of less-efficient mitochondria, we investigated mitophagy, a quality control process whereby defective mitochondria are removed through autophagy, usually seen in cells following damage or stress (Youle & Narendra 2011). This current study found no significant changes in the expression of genes involved in mitophagy including *Pink1, Park2* or *Tomm20.*Thus, it would appear as though factors which would normally trigger mitophagy in healthy cells are less likely to do so in cells which display features of a senescent cell type. Others have similarly reported that reduced mitophagy is a hallmark characteristic of senescent cells (Chapman *et al.* 2019). Given that many of the previously discussed parameters were indicative of a senescent phenotype, we assessed the expression of tumour suppressor gene *Tp53* which is a known marker of cell senescence. Surprisingly, *Tp53* expression decreased with gestational age in the placenta. It is important to note *Tp53* is regulated predominantly at the posttranslational level and that changes in activity link most closely with induction of a senescent phenotype (Rufini *et al.* 2013). Further studies are needed to investigate how placental Tp53 activity changes as pregnancy advances.

Mitochondrial dynamics consist of opposing processes that exist in equilibrium through fission and fusion. Under increased cellular stress, impaired mitochondrial fusion has been shown to be associated with pregnancy conditions, such as preeclampsia (Zhou *et al.* 2017). While this study did not measure mitofusin proteins in placentae complicated by pregnancy conditions, we found that as pregnancy progressed, there was a decrease in *Mfn1* and *2* gene expression and a decrease in MFN1 but an increase in MFN2 protein expression. This may be due to the nature of the mitofusin proteins, MFN1 is expressed in the outer mitochondrial membrane while MFN2 is expressed on the ER which we have not examined directly. Interestingly, previous studies have demonstrated that depletion of MFN1 results in the formation of highly fragmented smaller mitochondria, deletion of MFN2 results in the formation of larger mitochondrial fragments which cluster together into larger aggregates (Escobar-Henriques & Joaquim 2019). Additional studies on *Mfn1* mitochondrial mutant embryos caused disruption to the placental trophoblast giant cell layer; however, *Mfn2* mutant embryos did not alter placental development, which highlights the dynamic nature of these proteins during placental development (Chen *et al.* 2003). As mitochondria are responsible for energy production in all cells within animal species, there are variations between animal and human models. Although this study did not directly measure the changes within mouse and human models, previous research has identified bioenergetic differences in humans which largely reflect morphological differences within each cell type (Fisher *et al.* 2019*a*). Mitochondria from the human syncytiotrophoblast have small circular structures with reduced respiratory function and lower mitochondrial complex expression compared to mitochondria from the cytotrophoblast (Fisher *et al.* 2019*a*,*b*, Fisher * et al.*2020). It is likely that the changes across gestation identified in the current study reflect mitochondrial adaptions which occur as syncytialisation continues. The changes in MFN1 may therefore contribute to the morphological changes previously demonstrated.

As previously mentioned, mitochondria and ER have a bidirectional relationship; therefore, increases in ER stress can precede mitochondrial dysfunction during ageing (Chen *et al.* 2020). This study aimed to assess the relationship between mitochondrial genes and proteins associated with UPR as increases in mitochondrial dysfunction result in the unfolded protein response (Haynes *et al.* 2013). It was found that genes *Eif2ak3* and *Eif4g1* both decreased as gestation proceeded. In the absence of *Eif2AK3*, other studies have found cells to experience increased oxidative stress, leading to cell death (Ramnarayanan *et al.* 2016). Although this study did not measure markers of oxidative stress directly, we indirectly established increases in superoxide production across gestation through decreases in the antioxidant activity of SOD, which may indicate that there was an increase of ROS at E16.5. The mouse placenta consists of the maternal decidua, as well as the labyrinth, the main site of nutrient and gas exchange and the junctional zone, comprised of the main endocrine compartment of the placenta (Woods *et al.* 2018). Various studies have found differential expression of genes localised to either the labyrinth or the junctional zone. For example. when the translation initiation factor *Eif2s1* is deleted, a reduction in the labyrinth layer occurs, causing the fetus to be growth restricted (Yung *et al.* 2012). Unfortunately, this study identified specific genes and proteins in the whole placenta pooled from both labyrinth and junctional zone and did not determine if the expression was localised to a specific region within the placenta. Future research should determine if these genes and proteins are localised within the placenta at different periods of gestation.

## Conclusion

This study has demonstrated for the first time the expression patterns of mitochondrial proteins that regulate biogenesis, mitophagy, senescence, dynamics and UPR markers within the placenta across the second half of gestation in the mouse. We have clearly shown that mitochondria undergo changes in response to gestational progression and that pathways associated with cellular ageing seem to facilitate these adaptions in a healthy physiological pregnancy. This study holds great promise that mitochondrial markers across pregnancy may help to establish when a placenta is ageing inappropriately, albeit advanced ageing or advanced senescence. These findings may even provide a potential predictive mechanism to establish pregnancy pathologies such as fetal growth restriction or post-term stillbirth.

## Declaration of interest

The authors declare that there is no conflict of interest that could be perceived as prejudicing the impartiality of the research reported.

## Funding

This research did not receive any specific grant from any funding agency in the public, commercial or not-for-profit sector.

## Author contribution statement

L B performed all molecular, biochemical and protein assessment in this study. L B, J F, A P and J C conceptualised the project, provided intellectual input and were involved in writing and drafting of the manuscript. J C and S W generated all animal tissues. All authors approve the final submission of this manuscript.
